# Crystal structures of *N*-*tert*-butyl-3-(4-fluoro­phenyl)-5-oxo-4-[2-(tri­fluoro­meth­oxy)phen­yl]-2,5-di­hydro­furan-2-carboxamide and 4-(2*H*-1,3-benzodioxol-5-yl)-*N*-cyclo­hexyl-5-oxo-3-[4-(tri­fluoro­meth­yl)phen­yl]-2,5-di­hydro­furan-2-carboxamide

**DOI:** 10.1107/S2056989015000936

**Published:** 2015-01-24

**Authors:** Sue A. Roberts, Guillermo Martinez-Ariza, Christopher Hulme

**Affiliations:** a Chemistry and Biochemistry, University of Arizona, 1306 E. University Blvd, Tucson, AZ 85721, USA; b Department of Pharmacology and Toxicology, College of Pharmacy, University of Arizona, Tucson, AZ 85721, USA

**Keywords:** crystal structure, pharmaceuticals, butenolides, N—H⋯O hydrogen bonding

## Abstract

The title compounds, C_22_H_19_F_4_NO_4_, (I), and C_25_H_22_F_3_NO_5_, (II), each contain a central nearly planar di­hydro­furan­one ring. The r.m.s. deviation from planarity of these rings is 0.015 Å in (I) and 0.027 Å in (II). The mol­ecules are T-shaped, with the major conformational difference being the O—C—C—O torsion angle [−178.9 (1) in (I) and 37.7 (2)° in (II)]. In the crystal of (I), mol­ecules are linked by N—H⋯O hydrogen bonds, forming chains along [001] while in (II) mol­ecules are linked by N—H⋯O hydrogen bonds, forming chains along [010]. In (II), the tri­fluoro­methyl substituent is disordered over two sets of sites, with refined occupancies of 0.751 (3) and 0.249 (3).

## Abstract 2

The structures of two butenolide derivatives are reported. The conformations are differ largely in the orientation of the amide carbonyl atom.

## Chemical context   

Butenolides, also known as furan-2(5*H*)-ones or furan­ones, are a recurrent moiety in more than 13,000 natural products (De Souza, 2005 ▸) and possess different assorted biological applications, exemplified by cytotoxic (Jung *et al.*, 1990 ▸) and anti­biotic (Sikorska *et al.*, 2012 ▸) activities. Likewise, the butenolide derivative Vioxx® is a potent NSAID (non-steroidal anti-inflammatory drug) used for the relief of pain and inflammation (Prasit *et al.*, 1999 ▸) before it was withdrawn from the market in 2004. As a part of our scientific endeavors to access and mimic the complexity and diversity present in naturally occurring mol­ecular scaffolds, the title compounds were synthesized using a Passerini/Knoevenagel sequence and the crystal structures are reported herein. Other multi-component reaction-based approaches towards furan­ones have been reported, but they use limited starting materials. For example, they use unstable phospho­nates (Beck *et al.*, 2001 ▸), aliphatic substituents (Bossio *et al.*, 1993 ▸, 1994 ▸; Marcaccini *et al.*, 2000 ▸), or tricarbonyl inputs (Rossbach *et al.*, 2014 ▸).

## Structural commentary   

**Figure 1:**
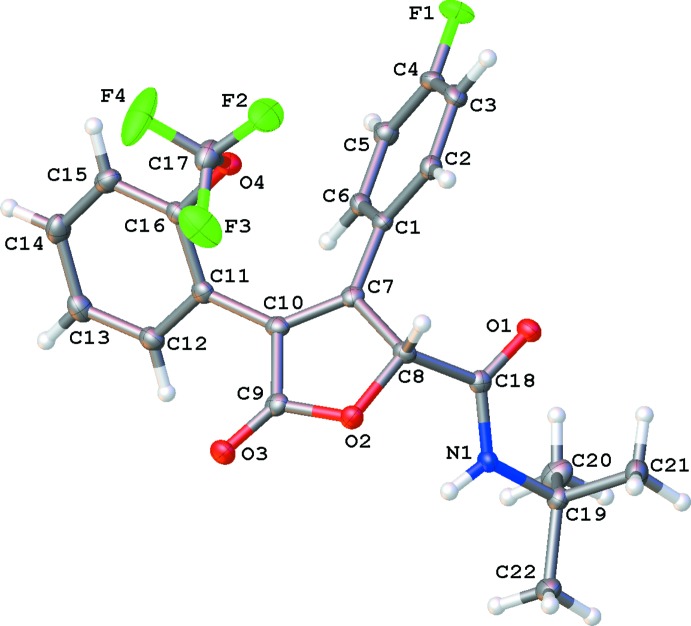
The mol­ecular structure of (I). Anisotropically refined atoms are shown as 50% probability displacement ellipsoids.

**Figure 2:**
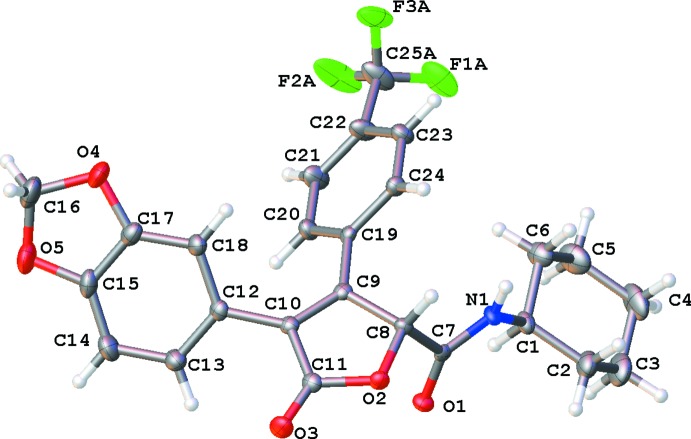
The mol­ecular structure of (II). The –CF_3_ substituent is disordered and only the major component is shown. Anisotropically refined atoms are shown as 50% probability displacement ellipsoids.

The mol­ecular structures of *N*-*tert*-butyl-3-(4-fluoro­phen­yl)-5-oxo-4-[2-(tri­fluoro­meth­oxy)phen­yl]-2,5-di­hydrofuran-2-carboxamide (I) (Fig. 1 ▸) and 4-(2*H*-1,3-benzodioxol-5-yl)-*N*-cyclo­hexyl-5-oxo-3-[4-(tri­fluoro­meth­yl)phen­yl]-2,5-di­hydrofuran-2-carboxamide (II) (Fig. 2 ▸) are similar. The mol­ecules are T-shaped, with the major conformational difference being the O1—C—C—O2 torsion angle. In (I), this torsion angle is −178.9 (1)°, whereas in (II), it is 37.7 (2)°.
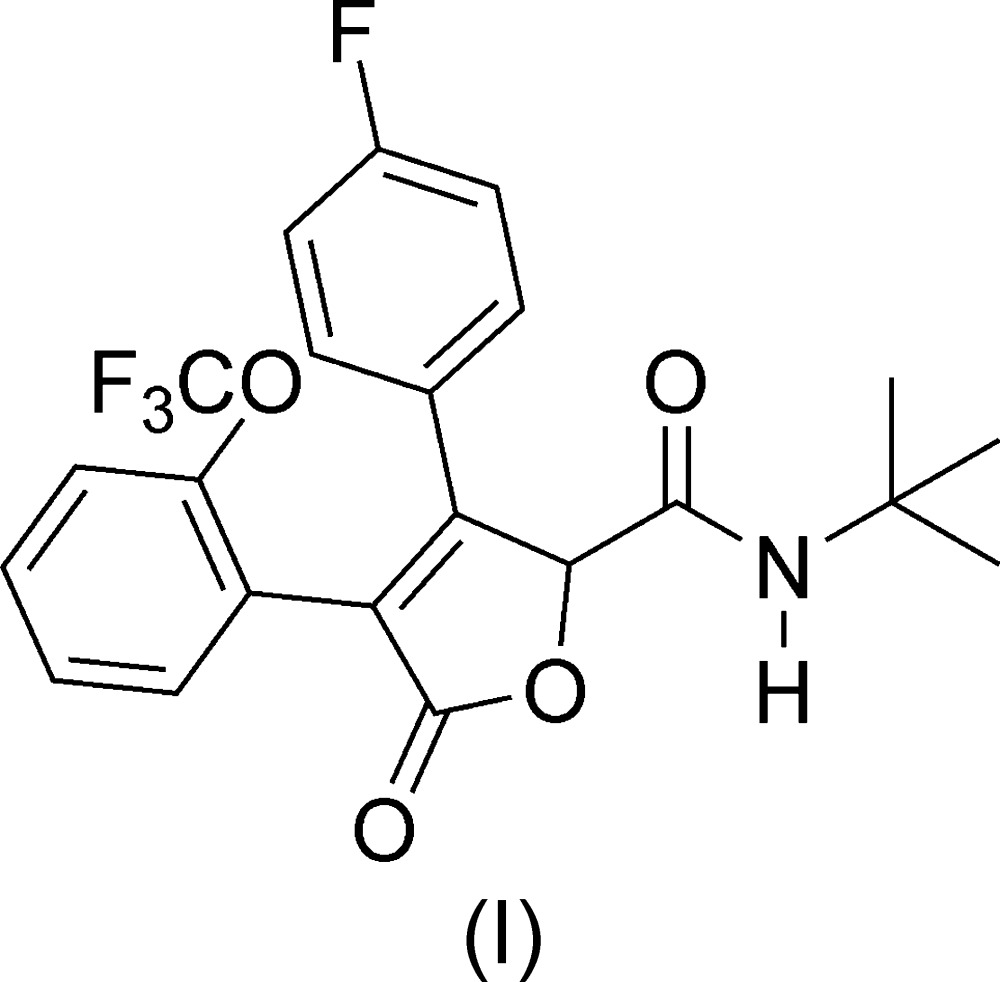
 
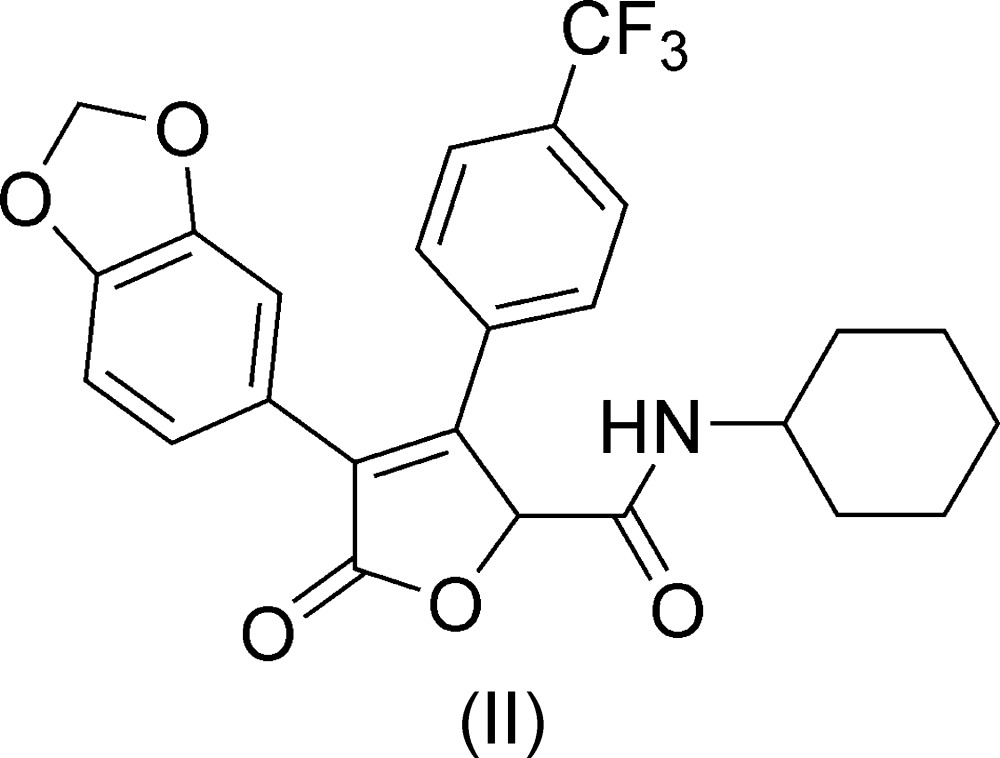


 In (II), the amide oxygen atom, O1, is tucked between O2 and H1*A*, with contact distances to O2 of 2.738 (1) Å and to H1*A* of 2.54 Å. The central, di­hydro­furan­one ring is nearly planar in both compounds. The r.m.s. deviation of these central rings is 0.015 Å in (I), and 0.027 Å in (II). In (I), the dihedral angle between the furan ring and the *p*-fluoro substituted benzene ring is 44.66 (4)° and with the tri­fluoro­meth­oxy-substituted benzene ring it is 48.71 (3)°. In (II), the dihedral angle between the furan ring and the *p*-tri­fluoro­methyl substituted benzene ring is 40.03 (5)° and the dihedral angle with the benzene ring of the benzo[1,3]dioxol-5-yl ring system is 43.06 (6)°. The cyclo­hexane ring of (II) is in a chair conformation.

The –CF_3_ substituent of (II) is disordered over two sets of sites. In the major component [occupancy = 0.751 (3)], F2*A* has a close contact to atom O1 [2.772 (2) Å] of a neighbouring molecule, which is lengthened to 3.093 (6) Å in the alternate configuration *i.e.* the minor component. Two hydrogen atoms from symmetry-equivalent mol­ecules flank F3*B* (H2*A*, 2.72 Å, and H23, 2.57 Å) and prevent rotational disorder from alleviating the close contact. In the minor component, the –CF_3_ group deviates from the plane of the aromatic ring, with C25*B* displaced by 0.36 (1) Å from the mean plane of the aromatic ring.

## Supra­molecular features   

**Figure 3:**
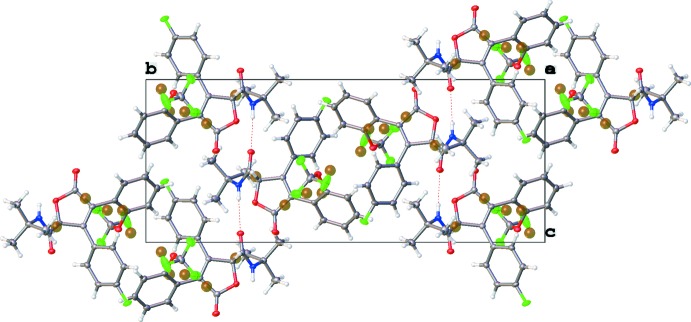
Part of the crystal structure of (I) viewed along [100]. The hydrogen bonds linking the mol­ecules into chains along [001] are shown as dotted lines.

**Figure 4:**
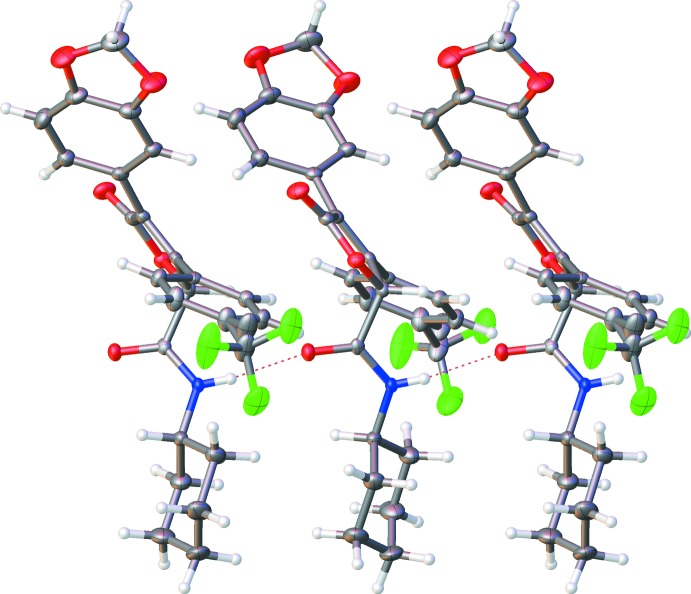
A hydrogen-bonded chain of mol­ecules of (II) propagating along [010]. The view is along the [100] direction. Hydrogen bonds are shown as dotted lines.

In both crystals, N—H⋯O hydrogen bonds connect the mol­ecules into chains which run, in (I) along the *c-*axis direction (Table 1 ▸), and in (II) along the *b-*axis direction (Table 2 ▸). The hydrogen-bonding graph set is *C*(4) in both (I) and (II). The partial packing plots of (I) (Fig. 3 ▸) and (II) (Fig. 4 ▸) illustrate the hydrogen-bonding motifs.

## Database survey   

The Cambridge Structural Database (CSD) contains few examples of 3,4,5-substituted furan-2(5*H*)-ones. A search (CSD Version 5.36, November 2014; Groom & Allen, 2014 ▸) found only one other structure with an amide attachment at the position 5 carbon, TIFXIP (Beck *et. al*, 2001). For TIFXIP, the O1—C—C—O2 torsion angles for the two mol­ecules in the asymmetric unit are −40.1 and 40.4°, similar to that found in (II), and the O1⋯O2 distances are 2.76 and 2.78 Å. When the search is expanded to include mol­ecules with a second organic substituent on the furan 5-carbon, additional structures are found. In six structures, where only one of the substituents is an amide, the O1—C—C—O2 torsion angle is 180° ± 30° (−150 to 150°); the value of −178.8 (1)° found for (I) falls in this range.

## Synthesis and crystallization   

**Compound (I)**: 4-fluoro­phenyl­glyoxal (1 eq., 0.5 mmol), tri­fluoro­meth­oxyphenyl­acetic acid (1 eq., 0.5 mmol) and *tert*-butyl isocyanide (1eq., 0.5 mmol) were dissolved in DCM (2 mL) and stirred at room temperature for 1 h. After confirming the exclusive formation of the Passerini product (*via* TLC and LC/MS), the solvent was removed and the crude product was dissolved in DMF (2 mL). Diiso­propyl­amine (DIPEA) (2 eq., 1 mmol, 140 µL) was added and the reaction mixture was heated at 393 K using microwave irradiation for 20 minutes. After cooling and verifying reaction completion (TLC and LC/MS), the crude mixture was directly purified by flash chromatography (EtOAc/hexane 0–100%) using an ISCO TM flash chromatography system to afford *N*-*tert*-butyl-3-(4-fluoro­phen­yl)-5-oxo-4-[2-(tri­fluoro­meth­oxy)phen­yl]-2,5-di­hydro­furan-2-carboxamide as a beige solid (67% yield).

**Compound (II)**: 4-tri­fluoro­methyl­phenyl­glyoxal (1 eq., 0.5 mmol), 3,4-methyl­ene­dioxy­phenyl­acetic acid (1eq., 0.5 mmol) and cyclo­hexyl isocyanide (1eq., 0.5 mmol) were dissolved in DCM (2 mL) and stirred at room temperature for 1 h. After confirming the exclusive formation of the Passerini product (*via* TLC and LC/MS), the solvent was removed and the crude product was dissolved in DMF (2 mL). Diiso­propyl­amine (DIPEA) (2 eq., 1 mmol, 140 µL) was added and the reaction mixture was heated at 393 K using microwave irradiation for 20 minutes. After cooling and verifying reaction completion (TLC and LC/MS), the crude mixture was directly purified by flash chromatography (EtOAc/hexane 0–100%) using an ISCO TM flash chromatography system to afford 4-(2*H*-1,3-benzodioxol-5-yl)-*N*-cyclo­hexyl-5-oxo-3-[4-(tri­fluoro­meth­yl)phen­yl]-2,5-di­hydro­furan-2-carboxamide as a yellow solid (61% yield).

For both compounds, crystals suitable for X-ray structure elucidation were obtained by slow evaporation of a solution of the compound in a mixture of ethyl acetate/hexa­nes (1:3).

## Refinement   

Crystal data, data collection and structure refinement details are summarized in Table 3 ▸. Hydrogen atoms were visible in the difference Fourier maps for both structures. The hydrogen atoms bonded to nitro­gen atoms which are involved in hydrogen bonding were placed at positions of the electron density peaks and freely refined. All other hydrogen atoms were placed at calculated positions and allowed to ride on their parent atoms: C—H = 0.98 Å for methyl H atoms and 0.95 Å for other H atoms, with *U*_iso_(H) = 1.5*U*_eq_(C) for methyl H atoms and = 1.2*U*_eq_(C) for other atoms.

In (II), the tri­fluoro­methyl substituent is disordered over two sets of sites with refined occupancies of 0.751 (3) and 0.249 (3). The disorder does not correspond to the expected rotational disorder of the –CF_3_ group, but rather consists of a deviation, in the minor component, of the central carbon atom out of the plane of the aromatic ring.

## Supplementary Material

Crystal structure: contains datablock(s) I, II, New_Global_Publ_Block. DOI: 10.1107/S2056989015000936/lh5745sup1.cif

Structure factors: contains datablock(s) I. DOI: 10.1107/S2056989015000936/lh5745Isup2.hkl

Structure factors: contains datablock(s) II. DOI: 10.1107/S2056989015000936/lh5745IIsup3.hkl

Supporting information file. DOI: 10.1107/S2056989015000936/lh5745Isup4.cml

Supporting information file. DOI: 10.1107/S2056989015000936/lh5745IIsup5.cml

CCDC references: 1043839, 1043838

Additional supporting information:  crystallographic information; 3D view; checkCIF report

## Figures and Tables

**Table 1:** Hydrogen-bond geometry (Å, °) for (I)

*D*—H⋯*A*	*D*—H	H⋯*A*	*D*⋯*A*	*D*—H⋯*A*
N1—H1⋯O1^i^	0.845 (17)	2.209 (17)	3.0098 (14)	158.2 (15)

Symmetry code: (i) 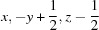.

**Table 2:** Hydrogen-bond geometry (Å, °) for (II)

*D*—H⋯*A*	*D*—H	H⋯*A*	*D*⋯*A*	*D*—H⋯*A*
N1—H1⋯O1^i^	0.821 (18)	2.061 (18)	2.8699 (16)	168.4 (16)

Symmetry code: (i) 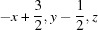.

**Table 3:** Experimental details

	(I)	(II)
Crystal data		
Chemical formula	C_22_H_19_F_4_NO_4_	C_25_H_22_F_3_NO_5_
*M*_r_	437.38	473.43
Crystal system, space group	Monoclinic, *P*2_1_/*c*	Orthorhombic, *P* *b* *c* *a*
Temperature (K)	100	100
*a*, *b*, *c* (Å)	8.0173 (8), 24.900 (2), 10.2186 (9)	19.2990 (7), 9.5345 (3), 24.2188 (7)
α, β, γ (°)	90, 96.738 (2), 90	90, 90, 90
*V* (Å^3^)	2025.9 (3)	4456.4 (2)
*Z*	4	8
Radiation type	Mo *K*α	Mo *K*α
μ (mm^−1^)	0.12	0.12
Crystal size (mm)	0.3 × 0.2 × 0.2	0.35 × 0.25 × 0.2

Data collection		
Diffractometer	Bruker APEXII CCD	Bruker APEXII CCD
Absorption correction	Multi-scan (*SADABS*; Bruker, 2009 ▸)	Multi-scan (*SADABS*; Bruker, 2012 ▸)
*T*_min_, *T*_max_	0.589, 0.746	0.609, 0.745
No. of measured, independent and observed [*I* > 2σ(*I*)] reflections	16716, 4139, 3698	36729, 3939, 3383
*R*_int_	0.022	0.033
(sin θ/λ)_max_ (Å^−1^)	0.625	0.595

Refinement		
*R*[*F* ^2^ > 2σ(*F* ^2^)], *wR*(*F* ^2^), *S*	0.034, 0.084, 1.03	0.035, 0.086, 1.05
No. of reflections	4139	3939
No. of parameters	287	324
No. of restraints	0	30
H-atom treatment	H atoms treated by a mixture of independent and constrained refinement	H atoms treated by a mixture of independent and constrained refinement
Δρ_max_, Δρ_min_ (e Å^−3^)	0.31, −0.32	0.27, −0.36

Computer programs: *APEX2* and *SAINT* (Bruker, 2009 ▸, 2012 ▸), *SHELXS97* (Sheldrick, 2008 ▸), *SHELXL2014* (Sheldrick, 2015 ▸), *OLEX2* (Dolomanov *et al.*, 2009 ▸) and *OLEX.SOLVE* (Bourhis *et al.*, 2015 ▸).

## supplementary crystallographic information

### (I) N-tert-Butyl-3-(4-fluorophenyl)-5-oxo-4-[2-(trifluoromethoxy)phenyl]-2,5-dihydrofuran-2-carboxamide Crystal data

**Table:**

C_22_H_19_F_4_NO_4_	*F*(000) = 904
*M**_r_* = 437.38	*D*_x_ = 1.434 Mg m^−^^3^
Monoclinic, *P*2_1_/*c*	Mo *K*α radiation, λ = 0.71073 Å
*a* = 8.0173 (8) Å	Cell parameters from 9193 reflections
*b* = 24.900 (2) Å	θ = 2.6–28.2°
*c* = 10.2186 (9) Å	µ = 0.12 mm^−^^1^
β = 96.738 (2)°	*T* = 100 K
*V* = 2025.9 (3) Å^3^	Prism, clear colourless
*Z* = 4	0.3 × 0.2 × 0.2 mm

### (I) N-tert-Butyl-3-(4-fluorophenyl)-5-oxo-4-[2-(trifluoromethoxy)phenyl]-2,5-dihydrofuran-2-carboxamide Data collection

**Table:**

Bruker APEXII CCD diffractometer	4139 independent reflections
Radiation source: sealed tube	3698 reflections with *I* > 2σ(*I*)
Graphite monochromator	*R*_int_ = 0.022
Detector resolution: 8 pixels mm^-1^	θ_max_ = 26.4°, θ_min_ = 1.6°
ω and φ scans	*h* = −9→10
Absorption correction: multi-scan (*SADABS*; Bruker, 2009)	*k* = −31→31
*T*_min_ = 0.589, *T*_max_ = 0.746	*l* = −12→12
16716 measured reflections	

### (I) N-tert-Butyl-3-(4-fluorophenyl)-5-oxo-4-[2-(trifluoromethoxy)phenyl]-2,5-dihydrofuran-2-carboxamide Refinement

**Table:**

Refinement on *F*^2^	Primary atom site location: structure-invariant direct methods
Least-squares matrix: full	Secondary atom site location: difference Fourier map
*R*[*F*^2^ > 2σ(*F*^2^)] = 0.034	Hydrogen site location: inferred from neighbouring sites
*wR*(*F*^2^) = 0.084	H atoms treated by a mixture of independent and constrained refinement
*S* = 1.03	*w* = 1/[σ^2^(*F*_o_^2^) + (0.0363*P*)^2^ + 1.005*P*] where *P* = (*F*_o_^2^ + 2*F*_c_^2^)/3
4139 reflections	(Δ/σ)_max_ = 0.001
287 parameters	Δρ_max_ = 0.31 e Å^−^^3^
0 restraints	Δρ_min_ = −0.32 e Å^−^^3^

### (I) N-tert-Butyl-3-(4-fluorophenyl)-5-oxo-4-[2-(trifluoromethoxy)phenyl]-2,5-dihydrofuran-2-carboxamide Special details

**Table:**

Experimental. Absorption correction: SADABS-2008/1 (Bruker, 2009) was used for absorption correction. wR2(int) was 0.0543 before and 0.0350 after correction. The Ratio of minimum to maximum transmission is 0.7899. The λ/2 correction factor is 0.0015.
Geometry. All e.s.d.'s (except the e.s.d. in the dihedral angle between two l.s. planes) are estimated using the full covariance matrix. The cell e.s.d.'s are taken into account individually in the estimation of e.s.d.'s in distances, angles and torsion angles; correlations between e.s.d.'s in cell parameters are only used when they are defined by crystal symmetry. An approximate (isotropic) treatment of cell e.s.d.'s is used for estimating e.s.d.'s involving l.s. planes.
Refinement. Refinement of *F*^2^ against ALL reflections. The weighted *R*-factor *wR* and goodness of fit *S* are based on *F*^2^, conventional *R*-factors *R* are based on *F*, with *F* set to zero for negative *F*^2^. The threshold expression of *F*^2^ > σ(*F*^2^) is used only for calculating *R*-factors(gt) *etc*. and is not relevant to the choice of reflections for refinement. *R*-factors based on *F*^2^ are statistically about twice as large as those based on *F*, and *R*- factors based on ALL data will be even larger.

### (I) N-tert-Butyl-3-(4-fluorophenyl)-5-oxo-4-[2-(trifluoromethoxy)phenyl]-2,5-dihydrofuran-2-carboxamide Fractional atomic coordinates and isotropic or equivalent isotropic displacement parameters (Å^2^)

**Table:**

	*x*	*y*	*z*	*U*_iso_*/*U*_eq_	
F1	0.41272 (11)	0.45346 (3)	0.87343 (7)	0.0292 (2)	
F2	−0.18072 (11)	0.38769 (4)	0.50333 (9)	0.0362 (2)	
O1	0.49555 (11)	0.26340 (4)	0.55486 (8)	0.01916 (19)	
O3	0.17815 (11)	0.32454 (4)	0.06255 (8)	0.0216 (2)	
O4	0.01276 (11)	0.43064 (4)	0.41800 (8)	0.0204 (2)	
F3	−0.16303 (13)	0.37653 (4)	0.29712 (9)	0.0449 (3)	
O2	0.23911 (11)	0.27391 (3)	0.24391 (8)	0.01759 (19)	
F4	−0.25704 (12)	0.45026 (4)	0.36505 (14)	0.0611 (4)	
N1	0.53587 (13)	0.23207 (4)	0.35123 (10)	0.0163 (2)	
C7	0.27384 (14)	0.34306 (5)	0.39881 (12)	0.0153 (2)	
C3	0.27935 (16)	0.38083 (5)	0.75683 (12)	0.0198 (3)	
H3	0.2340	0.3693	0.8339	0.024*	
C1	0.31213 (15)	0.37069 (5)	0.52612 (11)	0.0153 (2)	
C2	0.24707 (15)	0.35278 (5)	0.63949 (12)	0.0175 (3)	
H2	0.1803	0.3212	0.6360	0.021*	
C5	0.44905 (16)	0.44430 (5)	0.65007 (12)	0.0190 (3)	
H5	0.5197	0.4750	0.6555	0.023*	
C18	0.45164 (15)	0.25822 (5)	0.43641 (12)	0.0151 (2)	
C6	0.41299 (15)	0.41644 (5)	0.53279 (12)	0.0167 (2)	
H6	0.4576	0.4287	0.4560	0.020*	
C9	0.21170 (15)	0.32210 (5)	0.18045 (12)	0.0166 (2)	
C12	0.25614 (16)	0.44545 (5)	0.13706 (12)	0.0195 (3)	
H12	0.3302	0.4254	0.0898	0.023*	
C11	0.18971 (15)	0.42177 (5)	0.24354 (12)	0.0163 (2)	
C8	0.28161 (15)	0.28284 (5)	0.38281 (11)	0.0156 (2)	
H8	0.1925	0.2662	0.4304	0.019*	
C10	0.22884 (15)	0.36535 (5)	0.28033 (12)	0.0159 (2)	
C4	0.37925 (16)	0.42599 (5)	0.75857 (12)	0.0196 (3)	
C13	0.21580 (17)	0.49790 (5)	0.09903 (13)	0.0221 (3)	
H13	0.2616	0.5132	0.0260	0.027*	
C16	0.08092 (15)	0.45302 (5)	0.30855 (12)	0.0171 (2)	
C19	0.70069 (15)	0.20563 (5)	0.38884 (12)	0.0178 (3)	
C21	0.68968 (18)	0.16575 (6)	0.50104 (13)	0.0257 (3)	
H21A	0.6657	0.1851	0.5802	0.038*	
H21B	0.7967	0.1466	0.5194	0.038*	
H21C	0.5996	0.1399	0.4755	0.038*	
C15	0.04164 (16)	0.50550 (5)	0.27394 (13)	0.0210 (3)	
H15	−0.0303	0.5260	0.3222	0.025*	
C20	0.83339 (17)	0.24854 (6)	0.42715 (15)	0.0275 (3)	
H20A	0.8465	0.2711	0.3505	0.041*	
H20B	0.9407	0.2312	0.4576	0.041*	
H20C	0.7981	0.2708	0.4980	0.041*	
C14	0.10909 (17)	0.52781 (5)	0.16737 (13)	0.0231 (3)	
H14	0.0820	0.5637	0.1413	0.028*	
C17	−0.14429 (17)	0.41192 (6)	0.39516 (14)	0.0269 (3)	
C22	0.74529 (18)	0.17542 (6)	0.26798 (13)	0.0257 (3)	
H22A	0.6615	0.1474	0.2442	0.039*	
H22B	0.8564	0.1590	0.2878	0.039*	
H22C	0.7467	0.2005	0.1942	0.039*	
H1	0.496 (2)	0.2324 (6)	0.2709 (17)	0.025 (4)*	

### (I) N-tert-Butyl-3-(4-fluorophenyl)-5-oxo-4-[2-(trifluoromethoxy)phenyl]-2,5-dihydrofuran-2-carboxamide Atomic displacement parameters (Å^2^)

**Table:**

	*U*^11^	*U*^22^	*U*^33^	*U*^12^	*U*^13^	*U*^23^
F1	0.0373 (5)	0.0339 (5)	0.0166 (4)	−0.0029 (4)	0.0044 (3)	−0.0100 (3)
F2	0.0381 (5)	0.0359 (5)	0.0385 (5)	−0.0004 (4)	0.0206 (4)	0.0091 (4)
O1	0.0201 (4)	0.0233 (5)	0.0138 (4)	0.0021 (4)	0.0008 (3)	−0.0012 (3)
O3	0.0243 (5)	0.0250 (5)	0.0144 (4)	0.0033 (4)	−0.0023 (4)	−0.0031 (4)
O4	0.0208 (5)	0.0241 (5)	0.0172 (4)	0.0033 (4)	0.0067 (3)	0.0007 (4)
F3	0.0480 (6)	0.0522 (6)	0.0339 (5)	−0.0246 (5)	0.0014 (4)	−0.0021 (4)
O2	0.0184 (4)	0.0176 (4)	0.0157 (4)	0.0014 (3)	−0.0022 (3)	−0.0030 (3)
F4	0.0230 (5)	0.0470 (6)	0.1169 (10)	0.0145 (4)	0.0228 (6)	0.0425 (6)
N1	0.0167 (5)	0.0192 (5)	0.0124 (5)	0.0032 (4)	−0.0008 (4)	−0.0003 (4)
C7	0.0115 (5)	0.0179 (6)	0.0169 (6)	0.0009 (4)	0.0043 (4)	0.0000 (5)
C3	0.0215 (6)	0.0243 (6)	0.0143 (6)	0.0048 (5)	0.0055 (5)	0.0030 (5)
C1	0.0147 (6)	0.0173 (6)	0.0140 (6)	0.0045 (5)	0.0019 (4)	−0.0002 (4)
C2	0.0175 (6)	0.0174 (6)	0.0179 (6)	0.0019 (5)	0.0029 (5)	0.0025 (5)
C5	0.0187 (6)	0.0182 (6)	0.0198 (6)	0.0005 (5)	0.0009 (5)	−0.0015 (5)
C18	0.0151 (6)	0.0137 (5)	0.0162 (6)	−0.0018 (4)	0.0012 (4)	0.0007 (4)
C6	0.0167 (6)	0.0195 (6)	0.0142 (6)	0.0026 (5)	0.0028 (5)	0.0023 (5)
C9	0.0117 (6)	0.0195 (6)	0.0183 (6)	0.0026 (4)	0.0004 (4)	−0.0014 (5)
C12	0.0199 (6)	0.0233 (6)	0.0152 (6)	0.0002 (5)	0.0014 (5)	−0.0013 (5)
C11	0.0161 (6)	0.0189 (6)	0.0131 (6)	0.0001 (5)	−0.0017 (4)	−0.0017 (4)
C8	0.0145 (6)	0.0186 (6)	0.0135 (6)	−0.0008 (5)	0.0010 (4)	−0.0018 (5)
C10	0.0128 (6)	0.0202 (6)	0.0150 (6)	0.0009 (5)	0.0030 (4)	−0.0023 (5)
C4	0.0223 (6)	0.0233 (6)	0.0130 (6)	0.0051 (5)	0.0001 (5)	−0.0047 (5)
C13	0.0252 (7)	0.0234 (7)	0.0174 (6)	−0.0043 (5)	0.0007 (5)	0.0023 (5)
C16	0.0166 (6)	0.0211 (6)	0.0133 (6)	−0.0012 (5)	0.0002 (5)	−0.0009 (5)
C19	0.0155 (6)	0.0203 (6)	0.0172 (6)	0.0040 (5)	0.0009 (5)	0.0008 (5)
C21	0.0267 (7)	0.0273 (7)	0.0231 (7)	0.0074 (6)	0.0034 (5)	0.0070 (6)
C15	0.0207 (6)	0.0199 (6)	0.0217 (6)	0.0027 (5)	0.0002 (5)	−0.0040 (5)
C20	0.0171 (6)	0.0282 (7)	0.0370 (8)	0.0009 (5)	0.0020 (6)	−0.0023 (6)
C14	0.0270 (7)	0.0169 (6)	0.0241 (7)	−0.0008 (5)	−0.0025 (5)	0.0009 (5)
C17	0.0234 (7)	0.0245 (7)	0.0338 (8)	0.0037 (5)	0.0074 (6)	0.0079 (6)
C22	0.0255 (7)	0.0302 (7)	0.0214 (7)	0.0117 (6)	0.0030 (5)	−0.0008 (5)

### (I) N-tert-Butyl-3-(4-fluorophenyl)-5-oxo-4-[2-(trifluoromethoxy)phenyl]-2,5-dihydrofuran-2-carboxamide Geometric parameters (Å, º)

**Table:**

F1—C4	1.3578 (14)	C6—H6	0.9500
F2—C17	1.3217 (16)	C9—C10	1.4791 (17)
O1—C18	1.2271 (15)	C12—H12	0.9500
O3—C9	1.2047 (15)	C12—C11	1.3968 (17)
O4—C16	1.4154 (14)	C12—C13	1.3900 (18)
O4—C17	1.3373 (17)	C11—C10	1.4784 (17)
F3—C17	1.3294 (18)	C11—C16	1.3942 (17)
O2—C9	1.3694 (15)	C8—H8	1.0000
O2—C8	1.4376 (14)	C13—H13	0.9500
F4—C17	1.3257 (17)	C13—C14	1.3836 (19)
N1—C18	1.3326 (15)	C16—C15	1.3804 (18)
N1—C19	1.4860 (16)	C19—C21	1.5272 (17)
N1—H1	0.845 (17)	C19—C20	1.5262 (19)
C7—C1	1.4720 (16)	C19—C22	1.5240 (17)
C7—C8	1.5104 (16)	C21—H21A	0.9800
C7—C10	1.3423 (17)	C21—H21B	0.9800
C3—H3	0.9500	C21—H21C	0.9800
C3—C2	1.3855 (18)	C15—H15	0.9500
C3—C4	1.3795 (19)	C15—C14	1.3875 (19)
C1—C2	1.3982 (17)	C20—H20A	0.9800
C1—C6	1.3940 (17)	C20—H20B	0.9800
C2—H2	0.9500	C20—H20C	0.9800
C5—H5	0.9500	C14—H14	0.9500
C5—C6	1.3856 (17)	C22—H22A	0.9800
C5—C4	1.3768 (18)	C22—H22B	0.9800
C18—C8	1.5358 (17)	C22—H22C	0.9800
			
C17—O4—C16	116.25 (10)	F1—C4—C5	118.14 (12)
C9—O2—C8	109.78 (9)	C5—C4—C3	123.35 (12)
C18—N1—C19	123.44 (10)	C12—C13—H13	119.9
C18—N1—H1	118.0 (11)	C14—C13—C12	120.14 (12)
C19—N1—H1	118.4 (11)	C14—C13—H13	119.9
C1—C7—C8	123.57 (10)	C11—C16—O4	118.55 (11)
C10—C7—C1	127.58 (11)	C15—C16—O4	118.52 (11)
C10—C7—C8	108.85 (10)	C15—C16—C11	122.89 (11)
C2—C3—H3	120.9	N1—C19—C21	110.75 (10)
C4—C3—H3	120.9	N1—C19—C20	109.16 (10)
C4—C3—C2	118.12 (11)	N1—C19—C22	107.17 (10)
C2—C1—C7	121.58 (11)	C20—C19—C21	111.26 (11)
C6—C1—C7	119.01 (10)	C22—C19—C21	109.10 (11)
C6—C1—C2	119.40 (11)	C22—C19—C20	109.31 (11)
C3—C2—C1	120.41 (12)	C19—C21—H21A	109.5
C3—C2—H2	119.8	C19—C21—H21B	109.5
C1—C2—H2	119.8	C19—C21—H21C	109.5
C6—C5—H5	121.1	H21A—C21—H21B	109.5
C4—C5—H5	121.1	H21A—C21—H21C	109.5
C4—C5—C6	117.87 (12)	H21B—C21—H21C	109.5
O1—C18—N1	125.94 (11)	C16—C15—H15	120.6
O1—C18—C8	116.73 (10)	C16—C15—C14	118.81 (12)
N1—C18—C8	117.31 (10)	C14—C15—H15	120.6
C1—C6—H6	119.6	C19—C20—H20A	109.5
C5—C6—C1	120.82 (11)	C19—C20—H20B	109.5
C5—C6—H6	119.6	C19—C20—H20C	109.5
O3—C9—O2	121.49 (11)	H20A—C20—H20B	109.5
O3—C9—C10	130.18 (12)	H20A—C20—H20C	109.5
O2—C9—C10	108.33 (10)	H20B—C20—H20C	109.5
C11—C12—H12	119.4	C13—C14—C15	120.10 (12)
C13—C12—H12	119.4	C13—C14—H14	119.9
C13—C12—C11	121.13 (12)	C15—C14—H14	119.9
C12—C11—C10	120.68 (11)	F2—C17—O4	108.01 (12)
C16—C11—C12	116.91 (11)	F2—C17—F3	108.13 (12)
C16—C11—C10	122.36 (11)	F2—C17—F4	108.14 (12)
O2—C8—C7	104.62 (9)	F3—C17—O4	112.54 (11)
O2—C8—C18	112.83 (9)	F4—C17—O4	113.07 (12)
O2—C8—H8	108.5	F4—C17—F3	106.78 (13)
C7—C8—C18	113.81 (10)	C19—C22—H22A	109.5
C7—C8—H8	108.5	C19—C22—H22B	109.5
C18—C8—H8	108.5	C19—C22—H22C	109.5
C7—C10—C9	108.34 (11)	H22A—C22—H22B	109.5
C7—C10—C11	130.46 (11)	H22A—C22—H22C	109.5
C11—C10—C9	121.11 (11)	H22B—C22—H22C	109.5
F1—C4—C3	118.51 (11)		
			
O1—C18—C8—O2	−178.85 (10)	C12—C13—C14—C15	−0.2 (2)
O1—C18—C8—C7	62.13 (14)	C11—C12—C13—C14	0.44 (19)
O3—C9—C10—C7	−177.73 (13)	C11—C16—C15—C14	1.98 (19)
O3—C9—C10—C11	5.3 (2)	C8—O2—C9—O3	178.20 (11)
O4—C16—C15—C14	179.50 (11)	C8—O2—C9—C10	−2.52 (12)
O2—C9—C10—C7	3.08 (13)	C8—C7—C1—C2	−46.08 (17)
O2—C9—C10—C11	−173.85 (10)	C8—C7—C1—C6	135.23 (12)
N1—C18—C8—O2	−0.55 (15)	C8—C7—C10—C9	−2.32 (13)
N1—C18—C8—C7	−119.57 (11)	C8—C7—C10—C11	174.22 (12)
C7—C1—C2—C3	−177.29 (11)	C10—C7—C1—C2	134.48 (13)
C7—C1—C6—C5	178.60 (11)	C10—C7—C1—C6	−44.22 (18)
C1—C7—C8—O2	−178.70 (10)	C10—C7—C8—O2	0.83 (12)
C1—C7—C8—C18	−55.10 (15)	C10—C7—C8—C18	124.44 (11)
C1—C7—C10—C9	177.19 (11)	C10—C11—C16—O4	3.38 (17)
C1—C7—C10—C11	−6.3 (2)	C10—C11—C16—C15	−179.09 (12)
C2—C3—C4—F1	−179.44 (11)	C4—C3—C2—C1	−1.13 (18)
C2—C3—C4—C5	−0.42 (19)	C4—C5—C6—C1	−1.35 (18)
C2—C1—C6—C5	−0.12 (18)	C13—C12—C11—C10	177.90 (11)
C18—N1—C19—C21	55.13 (15)	C13—C12—C11—C16	0.44 (18)
C18—N1—C19—C20	−67.70 (15)	C16—O4—C17—F2	173.52 (10)
C18—N1—C19—C22	174.03 (11)	C16—O4—C17—F3	54.24 (15)
C6—C1—C2—C3	1.39 (18)	C16—O4—C17—F4	−66.86 (15)
C6—C5—C4—F1	−179.33 (11)	C16—C11—C10—C7	−48.09 (19)
C6—C5—C4—C3	1.65 (19)	C16—C11—C10—C9	128.08 (13)
C9—O2—C8—C7	1.13 (12)	C16—C15—C14—C13	−1.01 (19)
C9—O2—C8—C18	−123.11 (10)	C19—N1—C18—O1	−1.11 (19)
C12—C11—C10—C7	134.59 (14)	C19—N1—C18—C8	−179.23 (10)
C12—C11—C10—C9	−49.24 (16)	C17—O4—C16—C11	−100.23 (13)
C12—C11—C16—O4	−179.20 (11)	C17—O4—C16—C15	82.14 (14)
C12—C11—C16—C15	−1.67 (18)		

### (I) N-tert-Butyl-3-(4-fluorophenyl)-5-oxo-4-[2-(trifluoromethoxy)phenyl]-2,5-dihydrofuran-2-carboxamide Hydrogen-bond geometry (Å, º)

**Table:**

*D*—H···*A*	*D*—H	H···*A*	*D*···*A*	*D*—H···*A*
N1—H1···O1^i^	0.845 (17)	2.209 (17)	3.0098 (14)	158.2 (15)

Symmetry code: (i) *x*, −*y*+1/2, *z*−1/2.

### (II) 4-(2H-1,3-Benzodioxol-5-yl)-N-cyclohexyl-5-oxo-3-[4-(trifluoromethyl)phenyl]-2,5-dihydrofuran-2-carboxamide Crystal data

**Table:**

C_25_H_22_F_3_NO_5_	*D*_x_ = 1.411 Mg m^−^^3^
*M**_r_* = 473.43	Mo *K*α radiation, λ = 0.71073 Å
Orthorhombic, *P**b**c**a*	Cell parameters from 9996 reflections
*a* = 19.2990 (7) Å	θ = 2.5–25.6°
*b* = 9.5345 (3) Å	µ = 0.12 mm^−^^1^
*c* = 24.2188 (7) Å	*T* = 100 K
*V* = 4456.4 (2) Å^3^	Prism, clear colourless
*Z* = 8	0.35 × 0.25 × 0.2 mm
*F*(000) = 1968	

### (II) 4-(2H-1,3-Benzodioxol-5-yl)-N-cyclohexyl-5-oxo-3-[4-(trifluoromethyl)phenyl]-2,5-dihydrofuran-2-carboxamide Data collection

**Table:**

Bruker APEXII CCD diffractometer	3939 independent reflections
Radiation source: sealed tube	3383 reflections with *I* > 2σ(*I*)
Graphite monochromator	*R*_int_ = 0.033
Detector resolution: 8 pixels mm^-1^	θ_max_ = 25.0°, θ_min_ = 1.7°
φ and ω scans	*h* = −18→22
Absorption correction: multi-scan (*SADABS*; Bruker, 2012)	*k* = −11→11
*T*_min_ = 0.609, *T*_max_ = 0.745	*l* = −28→28
36729 measured reflections	

### (II) 4-(2H-1,3-Benzodioxol-5-yl)-N-cyclohexyl-5-oxo-3-[4-(trifluoromethyl)phenyl]-2,5-dihydrofuran-2-carboxamide Refinement

**Table:**

Refinement on *F*^2^	Primary atom site location: iterative
Least-squares matrix: full	Hydrogen site location: mixed
*R*[*F*^2^ > 2σ(*F*^2^)] = 0.035	H atoms treated by a mixture of independent and constrained refinement
*wR*(*F*^2^) = 0.086	*w* = 1/[σ^2^(*F*_o_^2^) + (0.0329*P*)^2^ + 2.7716*P*] where *P* = (*F*_o_^2^ + 2*F*_c_^2^)/3
*S* = 1.05	(Δ/σ)_max_ = 0.001
3939 reflections	Δρ_max_ = 0.27 e Å^−^^3^
324 parameters	Δρ_min_ = −0.36 e Å^−^^3^
30 restraints	

### (II) 4-(2H-1,3-Benzodioxol-5-yl)-N-cyclohexyl-5-oxo-3-[4-(trifluoromethyl)phenyl]-2,5-dihydrofuran-2-carboxamide Special details

**Table:**

Experimental. SADABS-2012/1 (Bruker,2012) was used for absorption correction. wR2(int) was 0.0555 before and 0.0466 after correction. The Ratio of minimum to maximum transmission is 0.8167. The λ/2 correction factor is 0.0015.
Geometry. All e.s.d.'s (except the e.s.d. in the dihedral angle between two l.s. planes) are estimated using the full covariance matrix. The cell e.s.d.'s are taken into account individually in the estimation of e.s.d.'s in distances, angles and torsion angles; correlations between e.s.d.'s in cell parameters are only used when they are defined by crystal symmetry. An approximate (isotropic) treatment of cell e.s.d.'s is used for estimating e.s.d.'s involving l.s. planes.

### (II) 4-(2H-1,3-Benzodioxol-5-yl)-N-cyclohexyl-5-oxo-3-[4-(trifluoromethyl)phenyl]-2,5-dihydrofuran-2-carboxamide Fractional atomic coordinates and isotropic or equivalent isotropic displacement parameters (Å^2^)

**Table:**

	*x*	*y*	*z*	*U*_iso_*/*U*_eq_	Occ. (<1)
O1	0.75295 (5)	0.55806 (10)	0.49296 (4)	0.0185 (2)	
O2	0.80402 (5)	0.43446 (10)	0.39940 (4)	0.0172 (2)	
O3	0.81211 (6)	0.58478 (12)	0.32882 (4)	0.0255 (3)	
O4	0.51011 (6)	0.45837 (13)	0.21882 (5)	0.0367 (3)	
F3B	0.4051 (13)	0.082 (2)	0.4558 (11)	0.0458 (10)	0.249 (3)
O5	0.52165 (7)	0.69044 (14)	0.19298 (5)	0.0384 (3)	
N1	0.72446 (7)	0.34318 (13)	0.52641 (5)	0.0165 (3)	
F1B	0.4084 (4)	0.2249 (7)	0.5233 (3)	0.0499 (6)	0.249 (3)
C7	0.74426 (7)	0.43101 (14)	0.48714 (6)	0.0140 (3)	
C19	0.61606 (7)	0.35359 (15)	0.41728 (5)	0.0144 (3)	
C8	0.74979 (7)	0.36625 (15)	0.42917 (5)	0.0145 (3)	
H8	0.7576	0.2627	0.4312	0.017*	
C9	0.68437 (7)	0.40034 (14)	0.39745 (6)	0.0144 (3)	
C24	0.60782 (8)	0.21905 (15)	0.43879 (6)	0.0173 (3)	
H24	0.6460	0.1561	0.4393	0.021*	
C11	0.77610 (8)	0.51128 (15)	0.35706 (6)	0.0175 (3)	
C20	0.55997 (8)	0.44519 (15)	0.41735 (6)	0.0199 (3)	
H20	0.5651	0.5371	0.4027	0.024*	
C12	0.65547 (8)	0.54436 (15)	0.31161 (6)	0.0173 (3)	
C10	0.70054 (7)	0.48558 (15)	0.35506 (6)	0.0156 (3)	
C17	0.56398 (8)	0.51889 (17)	0.24760 (6)	0.0244 (4)	
C15	0.57085 (9)	0.65775 (18)	0.23231 (6)	0.0268 (4)	
C23	0.54458 (8)	0.17701 (16)	0.45938 (6)	0.0217 (3)	
H23	0.5390	0.0847	0.4734	0.026*	
C14	0.61954 (9)	0.74357 (18)	0.25555 (7)	0.0295 (4)	
H14	0.6239	0.8392	0.2451	0.035*	
C22	0.48935 (8)	0.26896 (17)	0.45959 (7)	0.0261 (4)	
C13	0.66266 (8)	0.68364 (16)	0.29549 (6)	0.0230 (3)	
H13	0.6978	0.7394	0.3120	0.028*	
C1	0.69989 (8)	0.38852 (15)	0.58066 (6)	0.0198 (3)	
H1A	0.6828	0.4871	0.5770	0.024*	
C2	0.75650 (10)	0.38768 (19)	0.62406 (6)	0.0313 (4)	
H2A	0.7944	0.4515	0.6127	0.038*	
H2B	0.7759	0.2920	0.6276	0.038*	
C21	0.49677 (8)	0.40313 (17)	0.43866 (7)	0.0273 (4)	
H21	0.4586	0.4661	0.4389	0.033*	
C18	0.60503 (8)	0.45830 (16)	0.28706 (6)	0.0209 (3)	
H18	0.5997	0.3627	0.2973	0.025*	
C6	0.63867 (9)	0.29746 (18)	0.59757 (6)	0.0304 (4)	
H6A	0.6535	0.1981	0.5992	0.036*	
H6B	0.6016	0.3053	0.5695	0.036*	
C5	0.61007 (11)	0.3418 (2)	0.65399 (7)	0.0414 (5)	
H5A	0.5894	0.4366	0.6511	0.050*	
H5B	0.5731	0.2759	0.6654	0.050*	
C25B	0.4193 (8)	0.2164 (15)	0.4674 (5)	0.0359 (9)	0.249 (3)
C3	0.72705 (11)	0.4350 (2)	0.67971 (7)	0.0413 (5)	
H3A	0.7639	0.4307	0.7081	0.050*	
H3B	0.7113	0.5336	0.6768	0.050*	
C4	0.66675 (12)	0.3431 (2)	0.69726 (7)	0.0461 (5)	
H4A	0.6835	0.2462	0.7034	0.055*	
H4B	0.6476	0.3784	0.7326	0.055*	
C16	0.49374 (10)	0.5578 (2)	0.17675 (7)	0.0384 (5)	
H16A	0.4429	0.5649	0.1722	0.046*	
H16B	0.5141	0.5281	0.1411	0.046*	
F2B	0.3664 (3)	0.2895 (6)	0.4465 (4)	0.0671 (8)	0.249 (3)
C25A	0.4240 (2)	0.2222 (4)	0.48835 (13)	0.0359 (9)	0.751 (3)
F1A	0.43368 (11)	0.1921 (2)	0.54261 (8)	0.0499 (6)	0.751 (3)
F2A	0.37403 (9)	0.31660 (19)	0.48644 (14)	0.0671 (8)	0.751 (3)
F3A	0.4005 (4)	0.1032 (6)	0.4662 (3)	0.0458 (10)	0.751 (3)
H1	0.7263 (8)	0.2588 (19)	0.5199 (6)	0.016 (4)*	

### (II) 4-(2H-1,3-Benzodioxol-5-yl)-N-cyclohexyl-5-oxo-3-[4-(trifluoromethyl)phenyl]-2,5-dihydrofuran-2-carboxamide Atomic displacement parameters (Å^2^)

**Table:**

	*U*^11^	*U*^22^	*U*^33^	*U*^12^	*U*^13^	*U*^23^
O1	0.0213 (6)	0.0130 (5)	0.0212 (5)	−0.0007 (4)	0.0009 (4)	−0.0008 (4)
O2	0.0135 (5)	0.0206 (5)	0.0175 (5)	−0.0016 (4)	0.0016 (4)	0.0006 (4)
O3	0.0224 (6)	0.0336 (6)	0.0206 (6)	−0.0079 (5)	0.0041 (5)	0.0053 (5)
O4	0.0344 (7)	0.0465 (8)	0.0292 (6)	−0.0087 (6)	−0.0167 (5)	0.0072 (6)
F3B	0.0203 (12)	0.034 (2)	0.083 (3)	−0.0092 (14)	0.0007 (16)	0.0106 (14)
O5	0.0378 (7)	0.0447 (8)	0.0327 (7)	0.0069 (6)	−0.0141 (6)	0.0109 (6)
N1	0.0240 (7)	0.0106 (6)	0.0149 (6)	0.0013 (5)	−0.0005 (5)	−0.0006 (5)
F1B	0.0493 (13)	0.0453 (11)	0.0550 (13)	0.0014 (9)	0.0319 (10)	0.0147 (8)
C7	0.0101 (7)	0.0137 (7)	0.0182 (7)	0.0018 (6)	−0.0030 (6)	−0.0005 (6)
C19	0.0149 (7)	0.0175 (7)	0.0108 (6)	−0.0009 (6)	−0.0018 (6)	−0.0013 (5)
C8	0.0132 (7)	0.0139 (7)	0.0164 (7)	−0.0011 (6)	0.0023 (6)	0.0003 (6)
C9	0.0165 (7)	0.0127 (7)	0.0140 (7)	0.0001 (6)	−0.0005 (6)	−0.0041 (6)
C24	0.0149 (7)	0.0178 (7)	0.0191 (7)	0.0017 (6)	0.0001 (6)	0.0002 (6)
C11	0.0205 (8)	0.0181 (7)	0.0137 (7)	−0.0008 (6)	0.0021 (6)	−0.0024 (6)
C20	0.0198 (8)	0.0172 (7)	0.0226 (8)	0.0012 (6)	0.0005 (6)	0.0042 (6)
C12	0.0185 (8)	0.0209 (8)	0.0126 (7)	0.0003 (6)	0.0023 (6)	−0.0001 (6)
C10	0.0185 (8)	0.0151 (7)	0.0133 (7)	−0.0013 (6)	0.0011 (6)	−0.0028 (6)
C17	0.0214 (8)	0.0334 (9)	0.0185 (7)	−0.0024 (7)	−0.0018 (6)	−0.0002 (7)
C15	0.0261 (9)	0.0344 (9)	0.0201 (8)	0.0080 (7)	−0.0027 (7)	0.0059 (7)
C23	0.0209 (8)	0.0170 (8)	0.0273 (8)	−0.0013 (6)	0.0031 (7)	0.0030 (6)
C14	0.0353 (10)	0.0230 (8)	0.0303 (9)	0.0041 (7)	−0.0007 (8)	0.0073 (7)
C22	0.0163 (8)	0.0231 (8)	0.0388 (9)	−0.0007 (7)	0.0068 (7)	0.0018 (7)
C13	0.0271 (9)	0.0214 (8)	0.0204 (8)	−0.0020 (7)	−0.0011 (7)	−0.0003 (6)
C1	0.0297 (9)	0.0151 (7)	0.0144 (7)	0.0025 (6)	0.0009 (6)	0.0002 (6)
C2	0.0414 (11)	0.0311 (9)	0.0215 (8)	0.0004 (8)	−0.0080 (8)	0.0002 (7)
C21	0.0175 (8)	0.0232 (8)	0.0412 (10)	0.0052 (7)	0.0038 (7)	0.0036 (7)
C18	0.0241 (9)	0.0228 (8)	0.0157 (7)	−0.0019 (7)	−0.0003 (6)	0.0022 (6)
C6	0.0400 (10)	0.0301 (9)	0.0211 (8)	−0.0063 (8)	0.0055 (7)	−0.0023 (7)
C5	0.0561 (13)	0.0432 (11)	0.0248 (9)	−0.0085 (10)	0.0160 (9)	−0.0013 (8)
C25B	0.0282 (13)	0.0283 (11)	0.051 (3)	0.0034 (9)	0.023 (2)	0.008 (2)
C3	0.0624 (14)	0.0431 (11)	0.0183 (8)	0.0009 (10)	−0.0093 (8)	−0.0028 (8)
C4	0.0841 (16)	0.0385 (11)	0.0156 (8)	0.0002 (11)	0.0074 (9)	0.0039 (8)
C16	0.0328 (10)	0.0533 (12)	0.0290 (9)	0.0027 (9)	−0.0123 (8)	0.0072 (9)
F2B	0.0218 (7)	0.0383 (9)	0.141 (2)	0.0111 (7)	0.0384 (13)	0.0238 (13)
C25A	0.0282 (13)	0.0283 (11)	0.051 (3)	0.0034 (9)	0.023 (2)	0.008 (2)
F1A	0.0493 (13)	0.0453 (11)	0.0550 (13)	0.0014 (9)	0.0319 (10)	0.0147 (8)
F2A	0.0218 (7)	0.0383 (9)	0.141 (2)	0.0111 (7)	0.0384 (13)	0.0238 (13)
F3A	0.0203 (12)	0.034 (2)	0.083 (3)	−0.0092 (14)	0.0007 (16)	0.0106 (14)

### (II) 4-(2H-1,3-Benzodioxol-5-yl)-N-cyclohexyl-5-oxo-3-[4-(trifluoromethyl)phenyl]-2,5-dihydrofuran-2-carboxamide Geometric parameters (Å, º)

**Table:**

O1—C7	1.2310 (16)	C23—C22	1.380 (2)
O2—C8	1.4276 (17)	C14—H14	0.9500
O2—C11	1.3704 (17)	C14—C13	1.398 (2)
O3—C11	1.2008 (18)	C22—C21	1.383 (2)
O4—C17	1.3784 (19)	C22—C25B	1.455 (16)
O4—C16	1.427 (2)	C22—C25A	1.507 (4)
F3B—C25B	1.342 (17)	C13—H13	0.9500
O5—C15	1.3807 (19)	C1—H1A	1.0000
O5—C16	1.429 (2)	C1—C2	1.516 (2)
N1—C7	1.3234 (18)	C1—C6	1.522 (2)
N1—C1	1.4624 (18)	C2—H2A	0.9900
N1—H1	0.821 (18)	C2—H2B	0.9900
F1B—C25B	1.371 (11)	C2—C3	1.531 (2)
C7—C8	1.5375 (19)	C21—H21	0.9500
C19—C9	1.472 (2)	C18—H18	0.9500
C19—C24	1.394 (2)	C6—H6A	0.9900
C19—C20	1.391 (2)	C6—H6B	0.9900
C8—H8	1.0000	C6—C5	1.533 (2)
C8—C9	1.5131 (19)	C5—H5A	0.9900
C9—C10	1.346 (2)	C5—H5B	0.9900
C24—H24	0.9500	C5—C4	1.515 (3)
C24—C23	1.378 (2)	C25B—F2B	1.336 (14)
C11—C10	1.480 (2)	C3—H3A	0.9900
C20—H20	0.9500	C3—H3B	0.9900
C20—C21	1.384 (2)	C3—C4	1.518 (3)
C12—C10	1.476 (2)	C4—H4A	0.9900
C12—C13	1.391 (2)	C4—H4B	0.9900
C12—C18	1.405 (2)	C16—H16A	0.9900
C17—C15	1.381 (2)	C16—H16B	0.9900
C17—C18	1.369 (2)	C25A—F1A	1.358 (4)
C15—C14	1.367 (2)	C25A—F2A	1.321 (4)
C23—H23	0.9500	C25A—F3A	1.335 (6)
			
C11—O2—C8	109.46 (11)	N1—C1—C6	108.95 (12)
C17—O4—C16	104.46 (13)	C2—C1—H1A	107.7
C15—O5—C16	104.43 (13)	C2—C1—C6	111.71 (13)
C7—N1—C1	123.52 (12)	C6—C1—H1A	107.7
C7—N1—H1	118.0 (11)	C1—C2—H2A	109.7
C1—N1—H1	118.5 (11)	C1—C2—H2B	109.7
O1—C7—N1	125.44 (13)	C1—C2—C3	109.95 (15)
O1—C7—C8	119.35 (12)	H2A—C2—H2B	108.2
N1—C7—C8	114.96 (12)	C3—C2—H2A	109.7
C24—C19—C9	120.19 (13)	C3—C2—H2B	109.7
C20—C19—C9	120.48 (13)	C20—C21—H21	120.1
C20—C19—C24	119.25 (13)	C22—C21—C20	119.73 (14)
O2—C8—C7	109.22 (11)	C22—C21—H21	120.1
O2—C8—H8	111.3	C12—C18—H18	121.6
O2—C8—C9	104.91 (10)	C17—C18—C12	116.73 (14)
C7—C8—H8	111.3	C17—C18—H18	121.6
C9—C8—C7	108.63 (11)	C1—C6—H6A	109.4
C9—C8—H8	111.3	C1—C6—H6B	109.4
C19—C9—C8	121.10 (12)	C1—C6—C5	111.21 (14)
C10—C9—C19	129.72 (13)	H6A—C6—H6B	108.0
C10—C9—C8	108.88 (12)	C5—C6—H6A	109.4
C19—C24—H24	119.9	C5—C6—H6B	109.4
C23—C24—C19	120.27 (14)	C6—C5—H5A	109.4
C23—C24—H24	119.9	C6—C5—H5B	109.4
O2—C11—C10	108.87 (12)	H5A—C5—H5B	108.0
O3—C11—O2	120.71 (13)	C4—C5—C6	111.02 (17)
O3—C11—C10	130.42 (14)	C4—C5—H5A	109.4
C19—C20—H20	119.9	C4—C5—H5B	109.4
C21—C20—C19	120.30 (14)	F3B—C25B—F1B	103.5 (14)
C21—C20—H20	119.9	F3B—C25B—C22	119.5 (14)
C13—C12—C10	120.26 (13)	F1B—C25B—C22	104.5 (9)
C13—C12—C18	120.51 (14)	F2B—C25B—F3B	105.2 (16)
C18—C12—C10	119.23 (13)	F2B—C25B—F1B	103.1 (10)
C9—C10—C11	107.66 (13)	F2B—C25B—C22	118.8 (11)
C9—C10—C12	129.54 (14)	C2—C3—H3A	109.4
C12—C10—C11	122.78 (13)	C2—C3—H3B	109.4
O4—C17—C15	109.76 (14)	H3A—C3—H3B	108.0
C18—C17—O4	127.80 (15)	C4—C3—C2	111.16 (15)
C18—C17—C15	122.41 (15)	C4—C3—H3A	109.4
O5—C15—C17	109.59 (15)	C4—C3—H3B	109.4
C14—C15—O5	128.44 (15)	C5—C4—C3	111.39 (15)
C14—C15—C17	121.96 (15)	C5—C4—H4A	109.4
C24—C23—H23	120.0	C5—C4—H4B	109.4
C24—C23—C22	120.04 (14)	C3—C4—H4A	109.4
C22—C23—H23	120.0	C3—C4—H4B	109.4
C15—C14—H14	121.6	H4A—C4—H4B	108.0
C15—C14—C13	116.70 (15)	O4—C16—O5	107.95 (13)
C13—C14—H14	121.6	O4—C16—H16A	110.1
C23—C22—C21	120.41 (15)	O4—C16—H16B	110.1
C23—C22—C25B	120.0 (6)	O5—C16—H16A	110.1
C23—C22—C25A	117.36 (19)	O5—C16—H16B	110.1
C21—C22—C25B	117.6 (5)	H16A—C16—H16B	108.4
C21—C22—C25A	121.96 (19)	F1A—C25A—C22	113.3 (3)
C12—C13—C14	121.67 (15)	F2A—C25A—C22	113.2 (3)
C12—C13—H13	119.2	F2A—C25A—F1A	106.1 (3)
C14—C13—H13	119.2	F2A—C25A—F3A	108.4 (5)
N1—C1—H1A	107.7	F3A—C25A—C22	110.5 (4)
N1—C1—C2	112.81 (13)	F3A—C25A—F1A	104.8 (4)
			
O1—C7—C8—O2	37.65 (17)	C10—C12—C18—C17	179.02 (13)
O1—C7—C8—C9	−76.25 (16)	C17—O4—C16—O5	−19.09 (18)
O2—C8—C9—C19	−176.88 (12)	C17—C15—C14—C13	0.5 (2)
O2—C8—C9—C10	−2.55 (15)	C15—O5—C16—O4	18.95 (18)
O2—C11—C10—C9	2.98 (15)	C15—C17—C18—C12	−0.3 (2)
O2—C11—C10—C12	−175.42 (12)	C15—C14—C13—C12	−1.3 (2)
O3—C11—C10—C9	−176.73 (15)	C23—C22—C21—C20	0.0 (3)
O3—C11—C10—C12	4.9 (2)	C23—C22—C25B—F3B	−22.7 (17)
O4—C17—C15—O5	−0.23 (19)	C23—C22—C25B—F1B	92.3 (9)
O4—C17—C15—C14	178.50 (15)	C23—C22—C25B—F2B	−153.5 (8)
O4—C17—C18—C12	−178.10 (15)	C23—C22—C25A—F1A	59.2 (3)
O5—C15—C14—C13	178.93 (16)	C23—C22—C25A—F2A	−179.9 (2)
N1—C7—C8—O2	−147.74 (12)	C23—C22—C25A—F3A	−58.1 (5)
N1—C7—C8—C9	98.36 (14)	C13—C12—C10—C9	137.71 (16)
N1—C1—C2—C3	−179.57 (14)	C13—C12—C10—C11	−44.3 (2)
N1—C1—C6—C5	−179.20 (14)	C13—C12—C18—C17	−0.6 (2)
C7—N1—C1—C2	−95.91 (17)	C1—N1—C7—O1	9.6 (2)
C7—N1—C1—C6	139.43 (14)	C1—N1—C7—C8	−164.62 (13)
C7—C8—C9—C19	−60.19 (16)	C1—C2—C3—C4	56.9 (2)
C7—C8—C9—C10	114.14 (13)	C1—C6—C5—C4	−54.2 (2)
C19—C9—C10—C11	173.54 (14)	C2—C1—C6—C5	55.50 (19)
C19—C9—C10—C12	−8.2 (2)	C2—C3—C4—C5	−56.8 (2)
C19—C24—C23—C22	−1.1 (2)	C21—C22—C25B—F3B	141.0 (15)
C19—C20—C21—C22	−0.5 (2)	C21—C22—C25B—F1B	−103.9 (8)
C8—O2—C11—O3	175.07 (13)	C21—C22—C25B—F2B	10.3 (12)
C8—O2—C11—C10	−4.67 (14)	C21—C22—C25A—F1A	−114.8 (3)
C8—C9—C10—C11	−0.15 (15)	C21—C22—C25A—F2A	6.1 (4)
C8—C9—C10—C12	178.09 (13)	C21—C22—C25A—F3A	127.9 (4)
C9—C19—C24—C23	177.42 (13)	C18—C12—C10—C9	−41.9 (2)
C9—C19—C20—C21	−176.65 (14)	C18—C12—C10—C11	136.14 (15)
C24—C19—C9—C8	−41.76 (19)	C18—C12—C13—C14	1.4 (2)
C24—C19—C9—C10	145.21 (15)	C18—C17—C15—O5	−178.43 (15)
C24—C19—C20—C21	0.1 (2)	C18—C17—C15—C14	0.3 (3)
C24—C23—C22—C21	0.7 (3)	C6—C1—C2—C3	−56.43 (18)
C24—C23—C22—C25B	164.0 (5)	C6—C5—C4—C3	55.1 (2)
C24—C23—C22—C25A	−173.4 (2)	C25B—C22—C21—C20	−163.6 (5)
C11—O2—C8—C7	−111.87 (12)	C16—O4—C17—C15	11.93 (18)
C11—O2—C8—C9	4.42 (14)	C16—O4—C17—C18	−170.00 (17)
C20—C19—C9—C8	134.97 (14)	C16—O5—C15—C17	−11.56 (18)
C20—C19—C9—C10	−38.1 (2)	C16—O5—C15—C14	169.81 (18)
C20—C19—C24—C23	0.6 (2)	C25A—C22—C21—C20	173.8 (2)
C10—C12—C13—C14	−178.21 (14)		

### (II) 4-(2H-1,3-Benzodioxol-5-yl)-N-cyclohexyl-5-oxo-3-[4-(trifluoromethyl)phenyl]-2,5-dihydrofuran-2-carboxamide Hydrogen-bond geometry (Å, º)

**Table:**

*D*—H···*A*	*D*—H	H···*A*	*D*···*A*	*D*—H···*A*
N1—H1···O1^i^	0.821 (18)	2.061 (18)	2.8699 (16)	168.4 (16)

Symmetry code: (i) −*x*+3/2, *y*−1/2, *z*.
